# Supplementary material for: Identification of photoperiod-induced specific miRNAs in the adrenal glands of Sunite sheep (Ovis aries)
Source: Front Vet Sci. 2022 Jul 22;9:888207. doi: 10.3389/fvets.2022.888207 (PMC9354845; doi:10.3389/fvets.2022.888207)
Supplement: Supplementary file 1 [file Data_Sheet_1.ZIP › Supplementary file/miRDeep2 file.zip!/miRDeep2 file/Novel_Structure/SPLP42A3/Novel_542.pdf]

Provisional ID	:	NC_019477.2_32027
Score total	:	6.4
Score for star read(s)	:	3.9
Score for read counts	:	-0.8
Score for mfe	:	1.8
Score for randfold	:	1.6
Score for cons. seed	:	
Total read count	:	10
Mature read count	:	9
Loop read count	:	0
Star read count	:	1

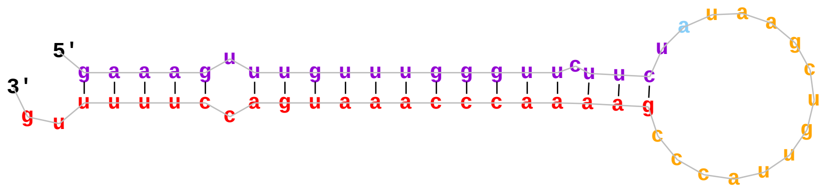

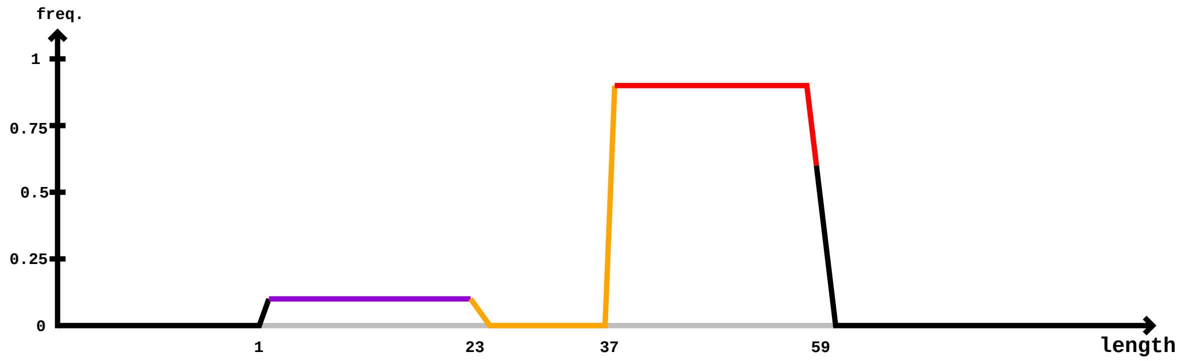


```
                         Star                                              Mature
5'- aaaagguauucgguuggccagaaaguuuguuuggguucuucuauaagcuguuacccgaaaacccaaaugaccuuuuuggccaacccaggauuaacauagcagaccugacugc   -3'   obs
    aaaagguauucgguuggccagaaaguuuguuuggguucuucuauaagcuguuacccgaaaacccaaaugaccuuuuuggccaacccaggauuaacauagcagaccugacugc         exp
    ...........((((((((((((((.(((((((((((.(((...............)))))))))))))).))))))))))))))).............((((......))))   reads   mm   sample
    ....................gaaaguuuguuuggguucuucu......................................................................   1       0    seq
    ........................................................gaaaacccaaaugaAcuuuuu...................................   3       1    seq
    ........................................................gaaaacccaaaugaAcuuuuug..................................   6       1    seq
```
